# CRISPR-Cas targeting in *Haloferax volcanii* promotes within-species gene exchange by triggering homologous recombination

**DOI:** 10.1093/femsml/uqaf047

**Published:** 2026-01-02

**Authors:** Deepak Kumar Choudhary, Israela Turgeman-Grott, Shachar Robinzon, Uri Gophna

**Affiliations:** The Shmunis School of Biomedicine and Cancer Research, George S. Wise Faculty of Life Sciences, Tel Aviv University, Tel Aviv, 69978, Israel; The Shmunis School of Biomedicine and Cancer Research, George S. Wise Faculty of Life Sciences, Tel Aviv University, Tel Aviv, 69978, Israel; The Shmunis School of Biomedicine and Cancer Research, George S. Wise Faculty of Life Sciences, Tel Aviv University, Tel Aviv, 69978, Israel; The Shmunis School of Biomedicine and Cancer Research, George S. Wise Faculty of Life Sciences, Tel Aviv University, Tel Aviv, 69978, Israel

**Keywords:** *Haloarchaea*, CRISPR-Cas, DNA repair, evolution, speciation, horizontal gene transfer

## Abstract

CRISPR (Clustered Regularly Interspaced Short Palindromic Repeats)-Cas (CRISPR-associated genes) systems provide adaptive immunity in bacteria and archaea against mobile genetic elements, but the role they play in gene exchange and speciation remains unclear. Here, we investigated how CRISPR-Cas targeting affects mating and gene exchange in the halophilic archaeon *Haloferax volcanii*. Surprisingly, we found that CRISPR-Cas targeting significantly increased mating efficiency between members of the same species, in contrast to its previously documented role in reducing interspecies mating. This enhanced mating efficiency was dependent on the Cas3 nuclease/helicase and extended beyond the targeted genomic regions. Further analysis revealed that CRISPR-Cas targeting promoted biased recombination in favor of the targeting strain (the strain containing the CRISPR-Cas system) during mating, resulting in an increased proportion of recombinant progeny that are positive for CRISPR-Cas. To test whether an increase in recombination is sufficient to increase mating efficiency, we tested whether strains lacking the Mre11–Rad50 complex, which are known to have elevated recombination activity, also exhibited higher mating success. Indeed, these strains showed higher mating, as did cells that were exposed to DNA damage using methyl methanesulfonate. These findings suggest that CRISPR-Cas systems in archaea play roles beyond their canonical immune function. They may contribute to speciation by facilitating within-species gene exchange while limiting between-species genetic transfer, thereby maintaining species boundaries.

## Teaser

CRISPR-Cas cutting of a genome allows increased recombination with the genome being cut, and thereby increases mating success.

## Introduction

CRISPR (Clustered Regularly Interspaced Short Palindromic Repeats)-Cas (CRISPR-associated genes) systems are now recognized as a predominant anti-viral defense that provides bacteria and archaea with adaptive immunity against mobile genetic elements (MGEs) (Sorek et al. 2008; Makarova et al., 2013). During infection, CRISPR-Cas systems target foreign DNA and acquire short sequences, called spacers, from invading genomes. These spacers are integrated into the CRISPR locus, serving as immune memory for targeting and preventing future infections (McGinn and Marraffini 2019).

CRISPR loci consist of short repetitive elements (repeats) interspersed with spacers, along with a set of *cas* genes (Hille and Charpentier 2016, McGinn and Marraffini 2019). Cas proteins serve multiple functions: some act as effector endonucleases that target and degrade foreign DNA that matches the system’s immune memory, others acquire new spacers, and some are responsible for crRNA maturation (Hille and Charpentier 2016, Maier et al., 2019). In type I systems, the effector protein Cas3 exhibits both helicase and nuclease activity (Maier et al., 2019, Miezner et al. 2023).

Halophilic archaea use a unique cell fusion-based mating process to exchange chromosomal and plasmid DNA within and between species (Naor et al. 2012, Naor and Gophna 2013). After the initial mating event, heterozygous cells are initially formed, containing genetic materials from both parents. This stage can later lead to the formation of two different types of progeny: recombinant cells, with chromosomal loci from both parental strains, and cells that may segregate and revert to the original (parental) genotypes (Naor and Gophna 2013). To study mating frequency, the selection of mating products is applied such that heterozygous cells will be able to grow, as well as recombinants with a favorable combination that contains the markers that are selected for, while cells that revert to the parental genotypes will be unable to grow.

Previous mating experiments between different species of the halophilic archeon *Haloferax* have shown that when the CRISPR-Cas systems of one species targets that of another, the efficiency of productive mating events is reduced compared to nontargeting mating (Turgeman-Grott et al. 2019). This raised the possibility that CRISPR-mediated degradation may cause mating cells to detach from their “offensive” mating partner prematurely, reducing the opportunity for gene exchange. Conversely, CRISPR-Cas mediated genome cleavage could actually stimulate HR during mating, due to the activity of the Cas3 nuclease/helicase of the type I-B CRISPR-Cas system (the most common system in halophilic archaea) (Maier et al., 2019, Miezner et al. 2023). This stimulation of HR is more likely to occur when homologous arms are perfectly identical, as typically observed within species (Naor et al. 2012, Turgeman-Grott et al. 2019).

In this study, we tested whether within-species CRISPR-Cas targeting affects mating success. Our results demonstrate that CRISPR-Cas *targeting* increases the frequency of gene exchange by influencing the mating process. Furthermore, we show that CRISPR-Cas targeting biased the recombination in favor of the targeting strain during mating, which could benefit CRISPR-Cas containing strains of *Haloferax* species, in which CRISPR-Cas is plasmid-encoded rather than ancestral. Importantly, CRISPR-Cas targeting can increase gene exchange within-species, while reducing between-species exchange, making these systems a factor that contributes to speciation.

## Results

### Within-species mating is increased by CRISPR-Cas targeting

Halophilic archaea can form cytoplasmic bridges that facilitate cell-to-cell contact, enabling genetic material transfer through a process called mating, which promotes horizontal gene transfer (HGT) (Naor and Gophna 2013; Mevarech et al., 1985, Rosenshine and Mevarech, 1991). Our lab previously demonstrated that CRISPR-Cas targeting reduces inter-species mating between *Haloferax volcanii* and a very genetically distant species *Haloferax mediterranei*, thereby limiting HGT (Turgeman-Grott et al. 2019). Interestingly, in that analysis, several natural strains had spacers that matched other strains from the same species (defined as having over 95% average nucleotide identity in coding genes, Supplementary Table S1), but not their own genomes. This implies that CRISPR-Cas spacer acquisition and potentially CRISPR-Cas targeting can occur both between and within species. Furthermore, when occurring within-species, targeting can be followed up by homologous recombination (HR)-based repair, which is very efficient when DNA identity is high (Naor et al. 2012). To further understand the role of CRISPR-Cas in gene exchange among halophilic archaea, we investigated its effect on within-species mating and recombination.

**Figure 1.**
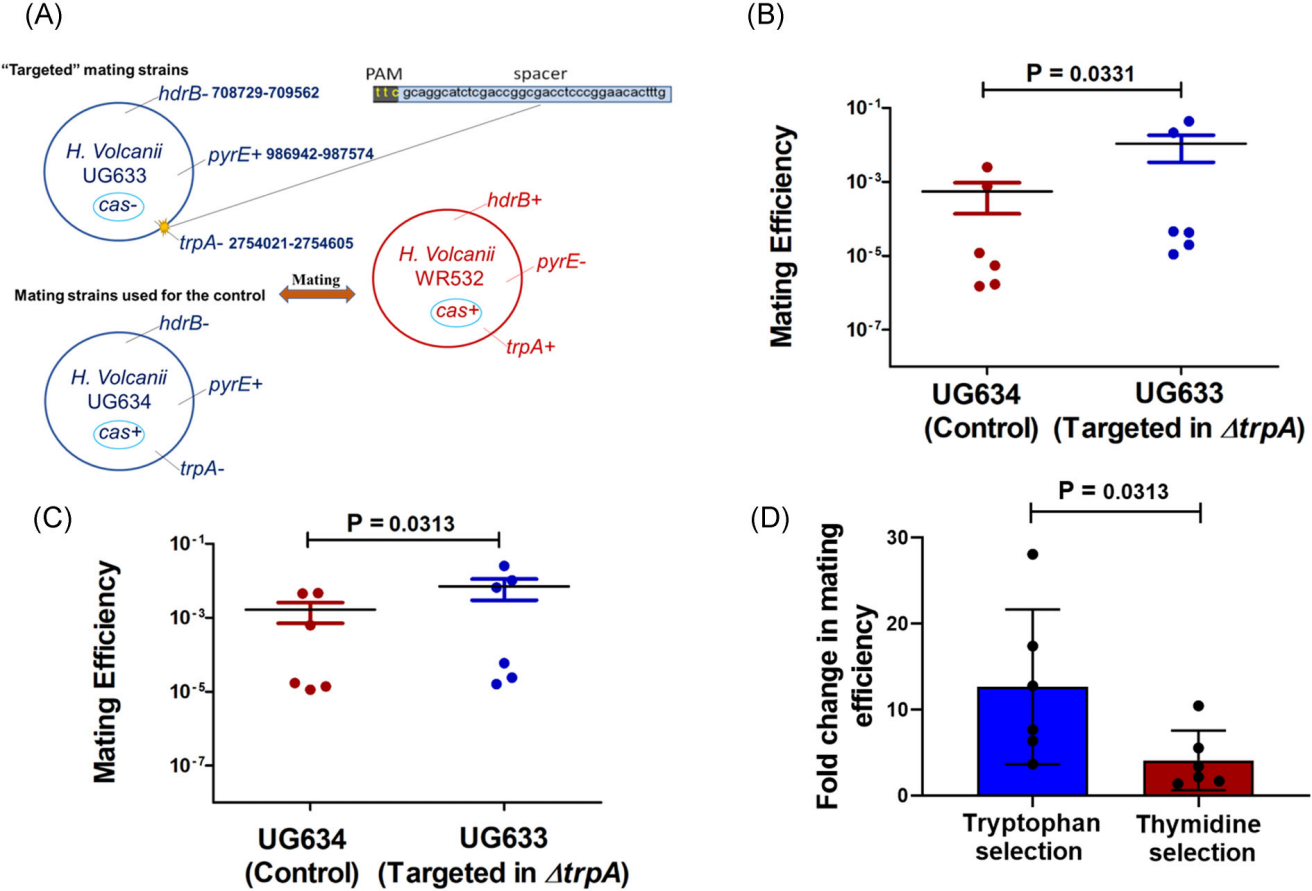
Within-species mating is increased by CRISPR-Cas targeting. (A) Strains used for the mating experiments. The “targeted” strain was engineered to contain a validated *H. volcanii* CRISPR spacer + PAM (40 bp)^13^ sequence so that *H. volcanii* CRISPR-Cas could target it during mating. The location of the spacer + PAM sequence insertion is highlighted and corresponds to the locus of the deleted *trpA* gene. The mating experiments were conducted between UG633 (*ΔhdrB, ΔtrpA, Δcas* genes, which also contains the target of the spacer inside *ΔtrpA*) and WR532 (*ΔpyrE*). The UG634 (*ΔhdrB, ΔtrpA*) nontargeted strain was used as control. Following mating, the cells were plated on Hv-Ca+ thymidine, and Hv-Ca+ tryptophan media. (B) Mating results after the selection of tryptophan. (C) Mating results after the selection of thymidine. The Wilcoxon matched-pairs signed-rank test was performed to compare targeted and nontargeted mating for both B and C, with *P* = 0.0331 and *P* = 0.0313, respectively (*N* = 6). (D) Comparison of mating efficiency between experiments using tryptophan (B) and thymidine (C) selection. The results shown are the mean of six independent experiments (*N* = 6). The Wilcoxon matched-pairs signed-rank test was performed for D with *P* = 0.0313.

We inserted a 40 bp spacer sequence into the *H. volcanii* genome. This sequence is targeted by a type I-B CRISPR-Cas system spacer that mediates DNA cleavage, known as interference (Maier et al., 2019, Fischer et al. 2012, Turgeman-Grott et al. 2019) (Supplementary Fig. 1). We then introduced this sequence into a strain lacking CRISPR-Cas genes to prevent self-targeting and autoimmunity. Next, we conducted mating experiments using WR532 (*ΔpyrE2*) that has the wild-type CRISPR-Cas system and two *H. volcanii* strains, either UG633 (*ΔhdrB, ΔtrpA*, and *Δcas* genes, containing the target spacer inside *ΔtrpA*) or UG634 (*ΔhdrB, ΔtrpA, cas+*) as a nontargeted control strain (Fig. 1A).

Surprisingly, within-species CRISPR-Cas targeting (UG633 mated with WR532) significantly increased mating efficiency (after the selection of the tryptophan marker) compared to the nontargeted control strain (UG634 mated with WR532) (Fig. 1B). Moreover, the same trend was observed when selecting for a different marker, thymidine (Fig. 1C).

Further, we investigated whether the increased mating efficiency was specifically due to CRISPR-Cas targeting rather than the presence of the CRISPR-Cas machinery itself, previously shown to affect recombination in *Haloferax* in the absence of targeting (Wörtz et al. 2022, Miezner et al. 2023). We conducted another mating experiment using two of the previously described strains: UG633 (*ΔhdrB, ΔtrpA*, and *Δcas* genes, containing the target of the spacer inside *ΔtrpA*) and WR532 (*ΔpyrE2*) with an active CRISPR-Cas system for this experiment, we used *H. volcanii* UG444 (*ΔhdrB* and *Δcas*) as a nontargeted control strain lacking the CRISPR-Cas system (Supplementary Fig. 2a). Consistent with our previous findings, CRISPR-Cas targeting (UG633 mated with WR532) increased mating efficiency by approximately fourfold compared to the nontargeted control (UG444 mated with WR532) strain (Supplementary Figure. 2b). These results suggest that the CRISPR-Cas targeting increased mating efficiency.

### CRISPR-Cas targeting affects gene exchange in loci distant from the targeted site

To test whether the effect of CRISPR-Cas targeting on mating success extends beyond the targeted region, i.e. beyond the CRISPR-Cas cut site, we selected for a distant genetic marker by plating the mating culture on a medium that selects for the presence of the *hdrB+* allele (using a medium containing *trpA+* and lacking *hdrB+*). The *hdrB* gene region is 801 881 bp distant from the targeted *trpA* site. Mating experiments were conducted between UG633 (*ΔhdrB, ΔtrpA*, and *Δcas* genes, with the target spacer inserted in *ΔtrpA*) and WR532 (*ΔpyrE2*) as shown in Fig. 1A. After mating, we first plated the culture on a medium containing thymidine and lacking tryptophan to select for cells containing the *trpA*+ marker. Interestingly, when we selected for the tryptophan marker, we observed high mating efficiency in CRISPR-Cas targeting compared to the nontargeted control (Fig. 1B).

To test whether the effect of CRISPR-Cas targeting extends beyond the targeted region, we plated the culture on a medium that selects for the *hdrB*+ marker (using a medium containing tryptophan but lacks thymidine). Surprisingly, CRISPR-Cas targeting still resulted in high mating efficiency compared to the nontargeted control (Fig. 1C), although this efficiency was approximately threefold lower than what was observed with the *trpA*- based selection (Fig. 1D). The increased mating efficiency was not restricted to the locus directly targeted by CRISPR-Cas (*trpA*+), suggesting broader promotion of gene exchange.

### Cas3 is required for increased mating in the targeted strain

Cas3 is a CRISPR-associated protein found in all Type I CRISPR-Cas systems, where it is critical for interference (elimination of foreign DNA) and exhibits both helicase and nuclease activity. Upon the invasion of foreign DNA, Cas3 is recruited by Cascade and cleaves spacer-matching DNA sequences processively, generating a single strand gap, which later becomes a double strand break (Maier et al., 2019, Sinkunas et al. 2011, Gong et al. 2014). Such gaps and breaks are expected to stimulate the DNA repair machinery of the cell, including the HR machinery. We tested whether Cas3 is required for the increased mating observed under targeted conditions. Specifically, if the increased mating is due to CRISPR-Cas interference, then deleting *cas3* in a mating partner with a CRISPR-Cas system that interferes with the target should reverse the increased mating phenotype.

**Figure 2.**
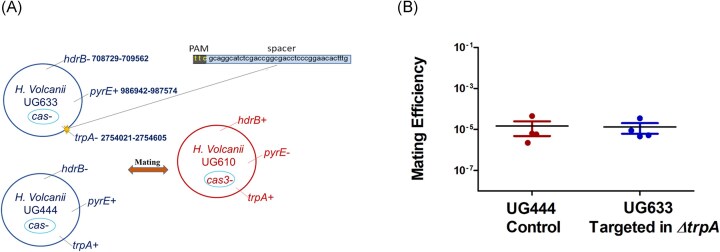
CRISPR-Cas activity is required for increased mating in the targeted strain. (A) The mating experiment has been conducted between UG633 (*ΔhdrB, ΔtrpA*, and *Δcas* genes, also contains the target of the spacer inside *ΔtrpA*) and UG610 (*ΔpyrE* and *Δcas3*). UG444 (*ΔhdrB* and *Δcas*) nontargeted strain has been used for the control. (B) Following mating, the cells were plated on Hv-Ca media. The targeted strain showed no change in mating efficiency in comparison to the control. The results shown are the mean of four independent experiments (*N* = 4). Wilcoxon matched-pairs signed-rank test, *P* = 1.0.

To test this, we chose a strain with only *cas3* deleted (but all other *cas* genes remain intact) and conducted a mating experiment between two sets of strains: UG633 *(ΔhdrB, ΔtrpA*, and *Δcas* genes, which also contains the target of the spacer within *ΔtrpA*) and UG610 (*ΔpyrE* and *Δcas3*). A nontargeted strain, UG444 (*ΔhdrB, Δcas*), was used as a control (Fig. 2A). After mating, the cells were plated on Hv-Ca plates, and unlike the previous experiment, no difference in mating was observed in the targeted strain (UG633 mated with UG610), compared with the nontargeted control (UG444 mated with WR532) (Fig. 2B). These findings suggest that the increased mating observed under targeted conditions is due to CRISPR-Cas-mediated interference and is dependent on Cas3 proteins.

### CRISPR-Cas systems drive biased recombination in favor of the targeting strain

CRISPR-induced genome cleavage has long been known to promote recombination between homologous chromosome arms and initiate HR in genome editing applications (Brunner et al. 2019, Zhang et al. 2020). As we have observed that CRISPR-Cas targeting increases within-species mating efficiency, it is plausible that this change in mating efficiency could be a result of an increased recombination rate. This is because presumably cells can enter a mating state, and then this state can be disrupted, but if recombination has already taken place, segregation will still result in the marker combination that is later selected for. However, CRISPR targeting may bias recombination because one mating partner introduces double-stranded breaks into the genome of the other.

**Figure 3.**
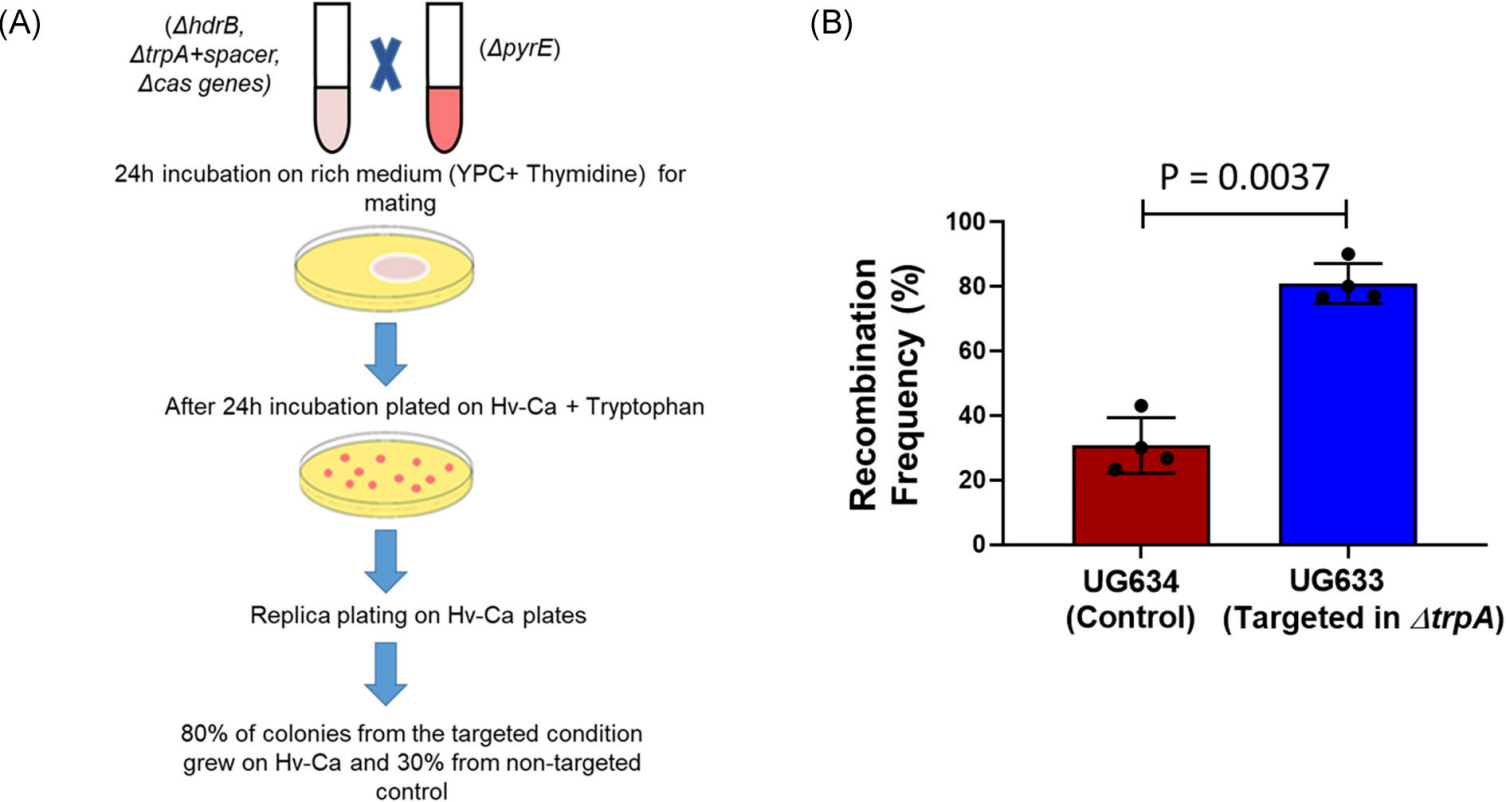
Recombination frequency is increased by CRISPR-Cas targeting. (A) Schematic of recombination frequency. (B) Recombination frequencies by CRISPR-Cas targeting in within-species mating. Recombination frequencies were calculated by replica-plating on un-supplemented Hv-Ca media and based on the PCR results, as described above in the result. The results shown are the mean of four independent experiments (*N* = 4). Paired *t*-test was performed to compare targeted and nontargeted mating with *P* = 0.0037.

To determine whether CRISPR targeting biases recombination indeed, we took 30–50 colonies (mating products) of the experiments described above (Fig. 1A and B), which were selected for the thymidine marker gene (from Hv-Ca +tryptophan plates). PCR screening for the *trpA* gene, which was not under selection, revealed that all mating products were recombinants rather than heterozygotes, as only either the *trpA^-^* or *trpA^+^* band was detected in each case, but never both (Supplementary Fig. 3). These recombinant mating products can be either WR532 cells that obtained the *pyrE* gene or UG633 cells that obtained the *hdrB* gene. We then performed replica plating onto Hv-Ca plates without additional supplementation on which only the former cells can grow because they are *trpA* (the *trpA* gene is located 801 881 bp away from the *hdrB* gene so for the UG633 to recombine both is far less likely). Approximately 80% of colonies from the targeted mating experiment grew on Hv-Ca plates, whereas only 30% of colonies from the control plate grew on Hv-Ca plates across all biological replicates (Fig. 3A and B), indicating that CRISPR targeting biases recombination, and favors gene acquisition by WR532.

Furthermore, when we took 50 colonies from the plate selected for the tryptophan marker from Hv-Ca +Thymidine plates (containing the target spacer inserted into *ΔtrpA*) and performed replica plating on Hv-Ca plates, bias (the directionality in genetic exchange) was even stronger, and all colonies grew on the Hv-Ca plates, probably because UG633 cells whose genome was cut by CRISPR-Cas in the *trpA* locus failed to acquire the *trpA* gene from WR532 cells. We conclude that CRISPR targeting between strains results in a genetic antagonism where the mating partner can provide genetic material to the “cutter” much more than vice versa, therefore benefiting strains that have CRISPR-Cas function.

### Disruption of *mre11* and *rad50* leads to increased mating efficiency in *H. volcanii*

**Figure 4.**
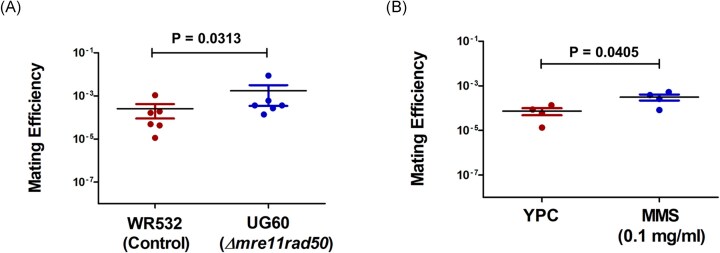
Disruption of *mre11* and *rad50* leads to increased mating efficiency in *H. volcanii*. (A) Effect of *mre11* and *radD50* deletion on archaeal mating frequency. The mating experiment was conducted between UG60 (*Δmre11, Δrad50, ΔpyrE)* and UG634 (*ΔhdrB, ΔtrpA*). The strain WR532 was used as a control. After mating, cells were plated on Hv-Ca+ tryptophan. *Mre11* and *rad50* deletion caused a significant increase in mating efficiency compared to the control. The results shown are the mean of six independent experiments (*N* = 6). The Wilcoxon matched-pairs signed-rank test was used to compare control (WR532) and *Δmre1, Δrad50* (UG60) deletion mating, with *P* = 0.0313. (B) MMS treatment leads to increased mating efficiency in *H. volcanii*. The mating experiments were conducted between the UG532 (*ΔpyrE*) and UG634 (*ΔhdrB, ΔtrpA*) strains. YPC plates with and without MMS (0.1 mg/ml) was used. The cultures were incubated on YPC+MMS plates and only YPC plates (used as a control) for 48 hours. The standard mating protocol was then followed. The results shown are the mean of four independent experiments (*N* = 4). The Wilcoxon matched-pairs signed-rank test was performed to compare MMS treated vs untreated control, with *P* = 0.0405.

Since within-species CRISPR-Cas targeting increased recombination efficiency as well as mating frequency, this suggests that increased HR is a force that will increase mating success. To assess if this is indeed a general mechanism not limited to CRISPR-Cas targeting, we tested whether an *mre11-rad50* knock-out strain, known to have higher HR activity than the wild type (Delmas et al. 2009, Miezner et al. 2023), will also show higher mating efficiency. We compared the mating efficiency of strain UG634 (*ΔhdrB, ΔtrpA*) with either strain UG60 (*Δmre11, Δrad50, ΔpyrE*) or strain UG532 (*ΔpyrE*) that has wild-type *mre11* and *rad50*. As expected, we observed higher mating efficiency when the mating partner was deleted for *mre11* and *rad50*. This supports the central role of HR in determining archaeal mating success (Fig. 4A).

We were then curious to investigate whether inducing double-strand DNA breaks using methyl methanesulfonate (MMS) could promote recombination in laboratory strains of haloarchaea. To address this hypothesis, we conducted a mating experiment (cultures were incubated during the fusion stage on YPC+MMS plates and YPC plates as a control for 48 hours) comparing strains with and without previous exposure to MMS. We tested two different concentrations of MMS: 0.1 and 0.2 mg/ml. Our results revealed that exposure to 0.1-mg/ml MMS significantly increased mating events by about fourfold (Fig. 4B). At the higher MMS concentration of 0.2 mg/ml, an increase in mating efficiency could also be observed. However, this was accompanied by high cell mortality and greater variability between experiments and should therefore be interpreted with caution (Supplementary Fig. 4). It is thus possible that an overly severe DNA damage no longer increases mating frequency. Overall, MMS exposure enhances mating efficiency, supporting the role of DNA damage in promoting higher mating.

## Discussion

HGT is considered a crucial driver of microbial evolution. Previous studies have indicated that CRISPR-Cas systems limit HGT through mechanisms such as conjugation and plasmid transformation, due to their ability to defend against foreign DNA acquisition (Marraffini and Sontheimer 2008). Previous work from our lab showed that CRISPR-Cas targeting can restrict gene exchange during inter-species mating between *H. volcanii* and *H. mediterranei* (Turgeman-Grott et al. 2019). However, the molecular mechanisms driving the interaction between CRISPR-Cas targeting and HGT within the same species have not been fully understood.

In this study, we demonstrate that CRISPR-Cas targeting significantly *increased* within-species mating efficiency in *H. volcanii*. This elevation of mating efficiency can be attributed to increased HR stimulated by CRISPR-Cas activity. Indeed, using a Δmre1 and Δrad50 strain with higher HR rates led to increased mating efficiency (Delmas et al. 2009, Miezner et al. 2023). The direct association between higher HR and higher mating can also explain the seeming contradiction between the results presented here and our previous work, where CRISPR-Cas targeting *reduced* inter-species mating efficiency between genetically distant *H. volcanii* and *H. mediterranei* (Turgeman-Grott et al. 2019). This discrepancy can be explained by the chances of successful HR being much higher when there is high sequence identity (within-species), as is long known for bacteria, eukaryotes, and archaea (Naor et al. 2012, Cadillo-Quiroz et al. 2012).

The increase in mating and recombination frequency was probably stimulated by the breaks that Cas3 generates during CRISPR-Cas-mediated DNA cleavage. This increase was dependent on the presence of Cas3 and a targeting CRISPR spacer. In agreement with a CRISPR-Cas stimulated process, recombination favored the integration of genetic material from the genome that was cut into that of the mating partner doing the cutting (CRISPR-Cas positive). Mechanistically, this is likely to be driven by a single strand of DNA from the lesion generated by Cas3 helicase-nuclease activity that invades the dsDNA of the CRISPR-positive mating partner. Nonetheless, increased recombination and mating were also observed far from the targeted site. This increase could be due to off-target effects caused by CRISPR-Cas activity in combination with the long recombination tracts often observed in *Haloferax* (Turgeman-Grott et al. 2019). Alternatively, HR could be increased globally due to the damage induced by CRISPR targeting. Indeed, DNA damage in haloarchaea has been shown to increase the expression of HR genes such as *radA* (Jones and Baxter, 2017, McCready et al. 2005).

The bias in recombination observed during targeting conditions is particularly intriguing. Our results show that CRISPR-Cas targeting leads to preferential gene flow from the targeted (UG 633) strain to the targeting (WR532) strain. This genetic antagonism could provide a competitive advantage to strains possessing active CRISPR-Cas systems, as they can acquire beneficial genetic material from related strains while limiting the reciprocal exchange. This asymmetric gene flow could affect adaptation and evolution within archaeal populations.

The fact that DNA cleavage increased overall mating rates prompted us to investigate whether less-specific DNA damage will also increase mating frequency. Indeed, exposure of *H. volcanii* to the DNA alkylating agent MMS in cells with intact *mre11*-*rad50* also increased mating frequency, but only about fourfold. This is perhaps to be expected, since in *H. volcanii* break repair is done primarily by microhomology mediated end-joining and not homologous recombination, in the presence of wild-type *mre11*-*rad50* (Delmas et al. 2009, Pérez-Arnaiz et al. 2020) (Fig. 4B). DNA repair by HR with an intact DNA template was also previously shown in the Sulfolobales, where UV exposure induced pili-mediated aggregation and subsequent genomic DNA exchange by the Ced system (Fröls et al. 2008, Ajon et al. 2011, Wolferen et al. 2016), a process somewhat analogous to haloarchaeal mating, though functionally distinct. While the results of our MMS experiment indicate that increased HR is the likely cause of increased mating, we cannot completely rule out additional effects of the mutagen, such as increasing cell permeability to DNA transfer.

In summary, the study presents evidence that CRISPR-Cas targeting boosts within-species mating efficiency in *H. volcanii* by enhancing HR rates. In haloarchaea, most CRISPR-Cas systems are encoded on megaplasmids, which can be horizontally transferred by mating. Thus, the contribution of CRISPR-Cas targeting to genetic barriers represents a rare example of a situation where MGEs promote speciation. Since CRISPR-Cas targeting between different species was shown to have the opposite effect (Turgeman-Grott et al. 2019), it follows that CRISPR-Cas systems help maintain species barriers in halophilic archaea. However, we acknowledge that such effects may not be generalizable to other microorganisms. Systems of other archaeal or bacterial species, especially those lacking the processive helicase-nuclease activity of Cas3, may not trigger similar DNA repair responses due to differences in their interference mechanisms. Moreover, DNA repair pathways and DNA repair preferences vary greatly between different microorganisms. The intriguing interplay between CRISPR-Cas, DNA repair, and HGT is nevertheless likely to prove important for the genome evolution of multiple archaea and bacteria.

## Material and methods

### Culture conditions

The wild-type *Haloferax* strains were routinely cultured at 45°C in either Hv-YPC or Hv-Ca medium. *Haloferax volcanii* transformants were selected and grown in either Hv-Ca or Hv-Enhanced Ca (Hv-ECa) medium. Thymidine (40 μg/ml) and tryptophan (50 μg/ml) were added when needed. Bacterial strains were cultured at 37°C in LB medium or in LB medium supplemented with ampicillin for strains carrying plasmids.

### Construction of *H. volcanii* strains with spacer+PAM sequence

The strain was constructed using the spacer+PAM sequence following the pop-in/pop-out protocol described in Bitan-Banin et al. (2003) and Allers et al. (2004). In this method, the *TrpA* flanking regions (upstream with HindIII and ApaI restriction sites) and a 40 bp spacer+PAM sequence (ttcgcaggcatctcgaccggcgacctcccggaacactttg) along with the *TrpA* flanking downstream regions (upstream with ApaI and EcorI restriction sites) are amplified by PCR using specific primer sets. These amplified regions are then cloned into the nonreplicating “suicide plasmid” pTA131, which harbors the *pyrE*2 selectable genetic marker.

The plasmids are transformed into *H. volcanii* mutant strains lacking *cas* genes, *hdrB*, and *pyrE2*. Transformants are selected on uracil-deficient media Hv-Ca (“pop-in”), where plasmids integrate into the chromosome. Subsequent counterselection on uracil and 5-FOA plates allows survival only of cells where integrated plasmids are excised through spontaneous intrachromosomal homologous recombination (“pop-out”). This process either restores the wild-type gene or achieves allele exchange. “Pop-out” strains are verified by PCR using primers flanking the deletion site and confirmed by Sanger sequencing. The parental strains and plasmids used in the constructions are detailed in Supplementary Tables S2 and S3.

### Mating protocol

The liquid cultures of both parental strains were grown separately until they reached the stationary phase (OD ∼1.1 to 1.3), as measured by optical density at 600 nm. The strains were then combined in a 1:1 ratio and filtered through 0.45-μm nitrocellulose filters using a Swinnex 25-mm filter holder. The filter containing the mating mixture was transferred to a rich medium plate (Hv-YPC supplemented with thymidine) and incubated for 48 hours at 45°C to allow for phenotypic expression. Following incubation, the cells were resuspended in Hv-Ca media and washed three times with the same media. Finally, the cell suspension was diluted and plated onto selective media chosen based on different mating markers. We followed the same protocol for all the mating experiments.

### MMS treatment assay

The mating experiment was conducted between strains UG532 (*ΔpyrE*) and UG634 (*ΔhdrB, ΔtrpA*). The membrane-containing cultures were incubated on both YPC+MMS (0.1 and 0.2 mg/ml) and YPC-only plates for 48 hours at 45°C, after which the standard mating protocol was followed.

### Measuring mating efficiencies

Mating efficiency was calculated by dividing the average number of colony-forming units (CFUs) on the selective mating plates by the average number of CFU of each parental strain grown on rich media plates.

### Average nucleotide identity calculation

Genomic relatedness between environmental isolates was determined using average nucleotide identity (ANI) calculations (http://www.ezbiocloud.net/tools/ani) (Yoon et al. 2017). Pairwise genome comparisons were performed to calculate the percentage of nucleotide identity across aligned genomic regions. This analysis provided species-level taxonomic classification, with ANI values ≥95% indicating strains of the same species.

## Supplementary Material

uqaf047_Supplemental_Files
