# Supplementary material for: CRISPR-Cas targeting in Haloferax volcanii promotes within-species gene exchange by triggering homologous recombination
Source: Microlife. 2026 Jan 2;7:uqaf047. doi: 10.1093/femsml/uqaf047 (PMC12814878; doi:10.1093/femsml/uqaf047)
Supplement: uqaf047_Supplemental_Files [file uqaf047_supplemental_files.zip › Supplementary Table 2.docx]

**Supplementary Table 2. List of all the strains and plasmids used in this study**

| **Archaeal strains & plasmids** | **Description** | **Source / Reference** |
| --- | --- | --- |
| WR540 | *H.volcanii ΔpyrE2, ΔhdrB, ΔtrpA* | ^30^ |
| WR532 | *H.volcanii ΔpyrE2* | ^30^ |
| UG438 | *∆cas genes* (all *CAS* genes +2 CRISPR locus)*, ∆hdrB, ∆pyrE2* | Lab strain |
| UG556 | *∆cas genes, ∆hdrB, ∆pyrE2, ∆TrpA*::+*H. volcanii* spacer+pam | This study |
| UG633 | *ΔhdrB, Δcas genes*, *∆TrpA*::+*H. volcanii* spacer+pam(ttcgcaggcatctcgaccggcgacctcccggaacactttg) based on UG438 | This study |
| UG634 | *ΔhdrB, ΔtrpA* | This study |
| UG444 | *∆cas genes* (all *CAS* genes +2 CRISPR locus)*, ∆hdrB* | Lab strain |
| UG610 | ΔpHV2*, ΔpyrE2, Δcas3*:213,632-214,258 | H26^b^ pUG523^c^ |
| UG60 | *Δmre11, rad51, ΔpyrE* | ^19^ |
| UG754 | *ΔhdrB+CAS3OE* | This study |
| UG754 | *ΔhdrB +* pTA927 | This study |
| **Plasmid** | **Description** | **Source / Reference** |
| pTA927 | Expression vector with *pyrE2* marker and pHV2 origin. | ^31^ |
| pTA131 | pBluescript II containing the *H. volcanii pyrE2* gene. | ^30^ |
| pUG545 | pTA131+TrpA (flanking up)+ spacer +TrpA (flanking down) | This Study |
| pUG921 | *CAS3OE* (cloned into pTA927) | This Study |

^b^ Parental strain.

^c^ Pop in plasmid used for strain generation
