# Supplementary material for: CRISPR-Cas targeting in Haloferax volcanii promotes within-species gene exchange by triggering homologous recombination
Source: Microlife. 2026 Jan 2;7:uqaf047. doi: 10.1093/femsml/uqaf047 (PMC12814878; doi:10.1093/femsml/uqaf047)
Supplement: uqaf047_Supplemental_Files [file uqaf047_supplemental_files.zip › Supplementary Table 3.docx]

**Supplementary Table 3. List of oligonucleotides used in this study**

| **Primer** | **Sequence (5’→ 3’)** | **Description** |
| --- | --- | --- |
| SR93 | AAAAAAGCTTCGAGCATGGTTCGGGACTT | *ΔTrpA* upstream forward primer, HindIII site is underline, for generating *TrpA-KO* with spacer*.* |
| SR94 | AAAAGGGCCCCGTCTTCGAGCGACATCAC | *ΔTrpA* upstream reverse primer, ApaI site is underline, for generating *TrpA-KO* with spacer*.* |
| SR95 | AAAAGGGCCCttcgcaggcatctcgaccggcgacctcccggaacactttg  AACGACTCACCGACTACGAC | *ΔTrpA* downstream forward primer, ApaI site is underline and spacer sequence in small letter, for generating *TrpA-KO* with spacer*.* |
| SR96 | AAAAGAATTCCGAGCCGACGTTGATATGG | *ΔTrpA* downstream forward primer, EcorI site is underline, for generating *TrpA-KO* with spacer*.* |
| SR105 | TGTAAAACGACGGCCAGTGA | Forward primer to verify successful cloning of the TrpA flanking sequence and spacer into pTA131 |
| SR106 | ACTTTATGCTTCCGGCTCGT | Reverse primer to verify successful cloning of the TrpA flanking sequence and spacer into pTA131 |
| IS229 | GGGGTACCGGGGCCTCTAATCGACGTAGGC | Forward primer to insert *pyrE gene* into *H. volcanii* |
| IS230 | GCCAAGCTTGTGCCTATTTCTACGTCACC | Reverse primer to insert *pyrE gene* into *H. volcanii* |
