# Supplementary material for: CRISPR-Cas targeting in Haloferax volcanii promotes within-species gene exchange by triggering homologous recombination
Source: Microlife. 2026 Jan 2;7:uqaf047. doi: 10.1093/femsml/uqaf047 (PMC12814878; doi:10.1093/femsml/uqaf047)
Supplement: uqaf047_Supplemental_Files [file uqaf047_supplemental_files.zip › Supplementary Table S1.docx]

**Supplementary Table S1.**

| **Targeting/targeted** | **ANI %** | **Targeting spacers** |
| --- | --- | --- |
| **24N-> 24N** | **100** | **1** |
| **24N -> 48N** | **99.45** | **4** |
| **24N-> 47N** | **99.45** | **1** |
| **24N-> 19N** | **67.91** | **4** |
| **47N-> 47N** | **100** | **1** |
| **47N-> 48N** | **99.65** | **4** |
| **47N-> 24N** | **99.45** | **1** |
| **47N-> 19N** | **69.43** | **2** |
| **48N-> 19N** | **91.44** | **0** |
| **48N-> 48N** | **100** | **0** |
| **48N-> 24N** | **99.45** | **0** |
| **48N-> 47N** | **99.65** | **1** |
| **19N-> 19N** | **100** | **0** |
| **19N-> 48N** | **91.44** | **0** |
| **19N-> 47N** | **69.43** | **1** |
| **19N-> 24N** | **67.91** | **0** |
