# Supplementary figures and images for: CRISPR-Cas targeting in Haloferax volcanii promotes within-species gene exchange by triggering homologous recombination
Source: Microlife. 2026 Jan 2;7:uqaf047. doi: 10.1093/femsml/uqaf047 (PMC12814878; doi:10.1093/femsml/uqaf047)

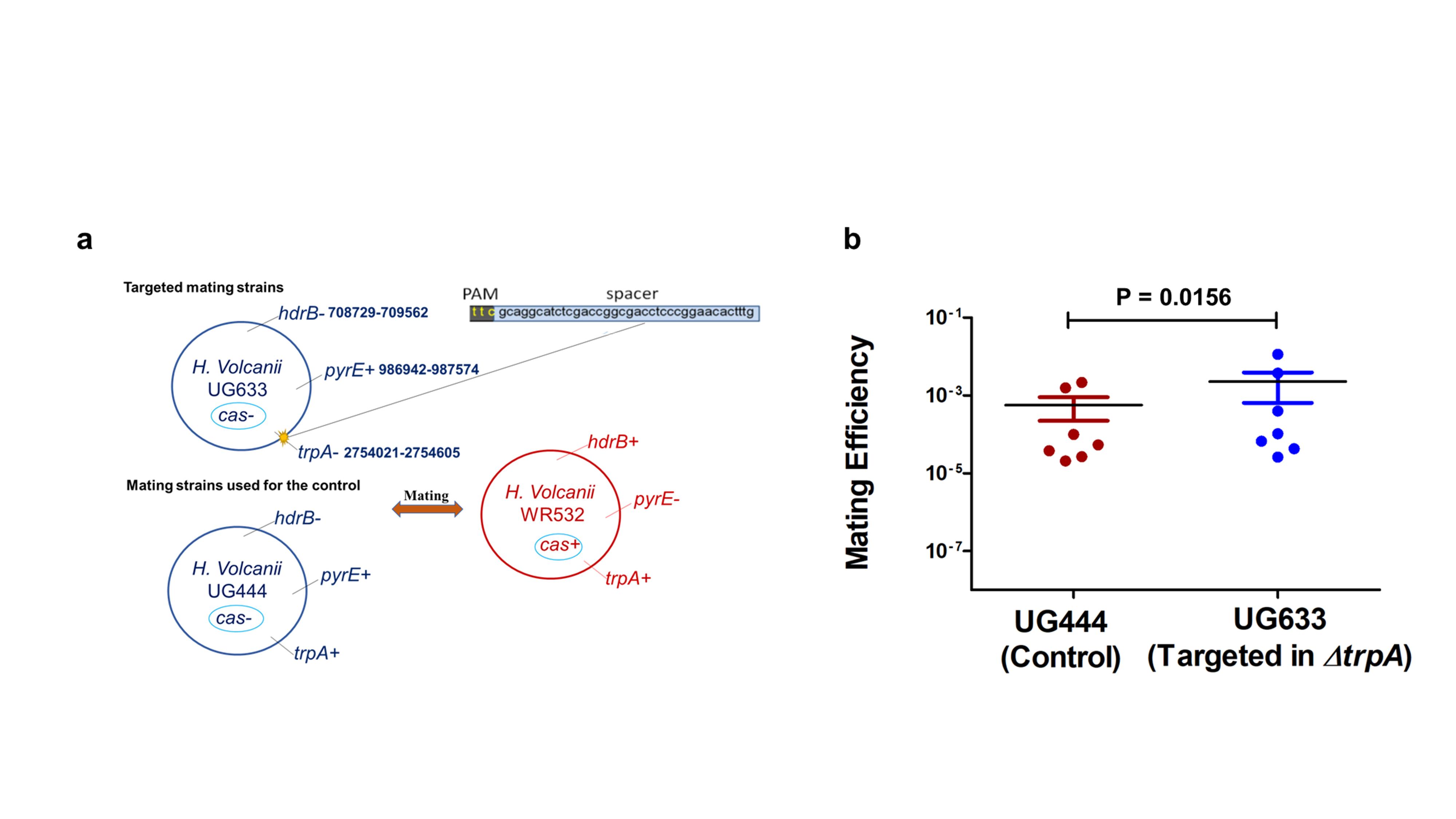

Supplement: uqaf047_Supplemental_Files [file uqaf047_supplemental_files.zip › 06-11-25- Supplementary Fig. 2 .tif]

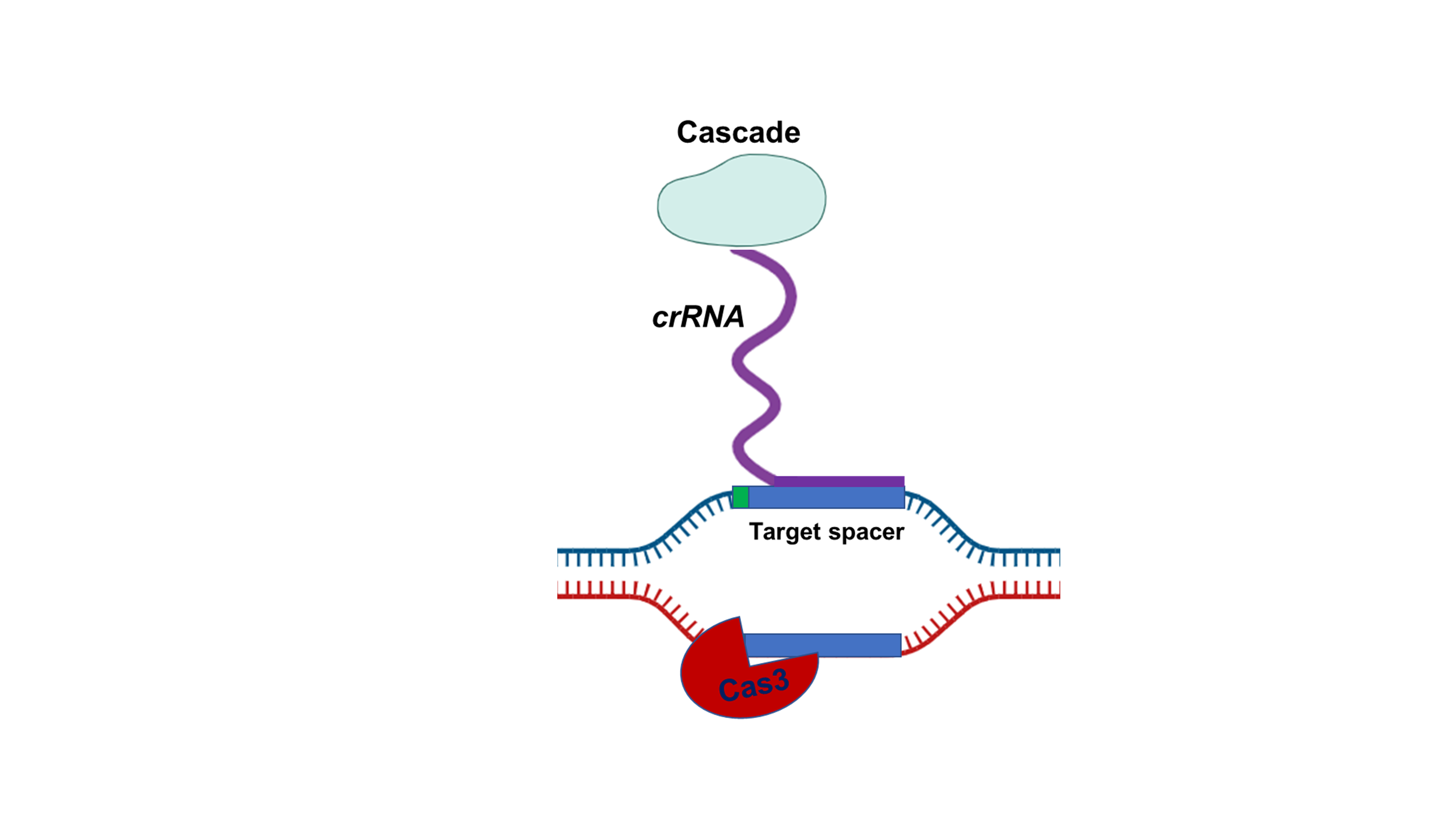

Supplement: uqaf047_Supplemental_Files [file uqaf047_supplemental_files.zip › Supplementary Fig. 1 .tif]

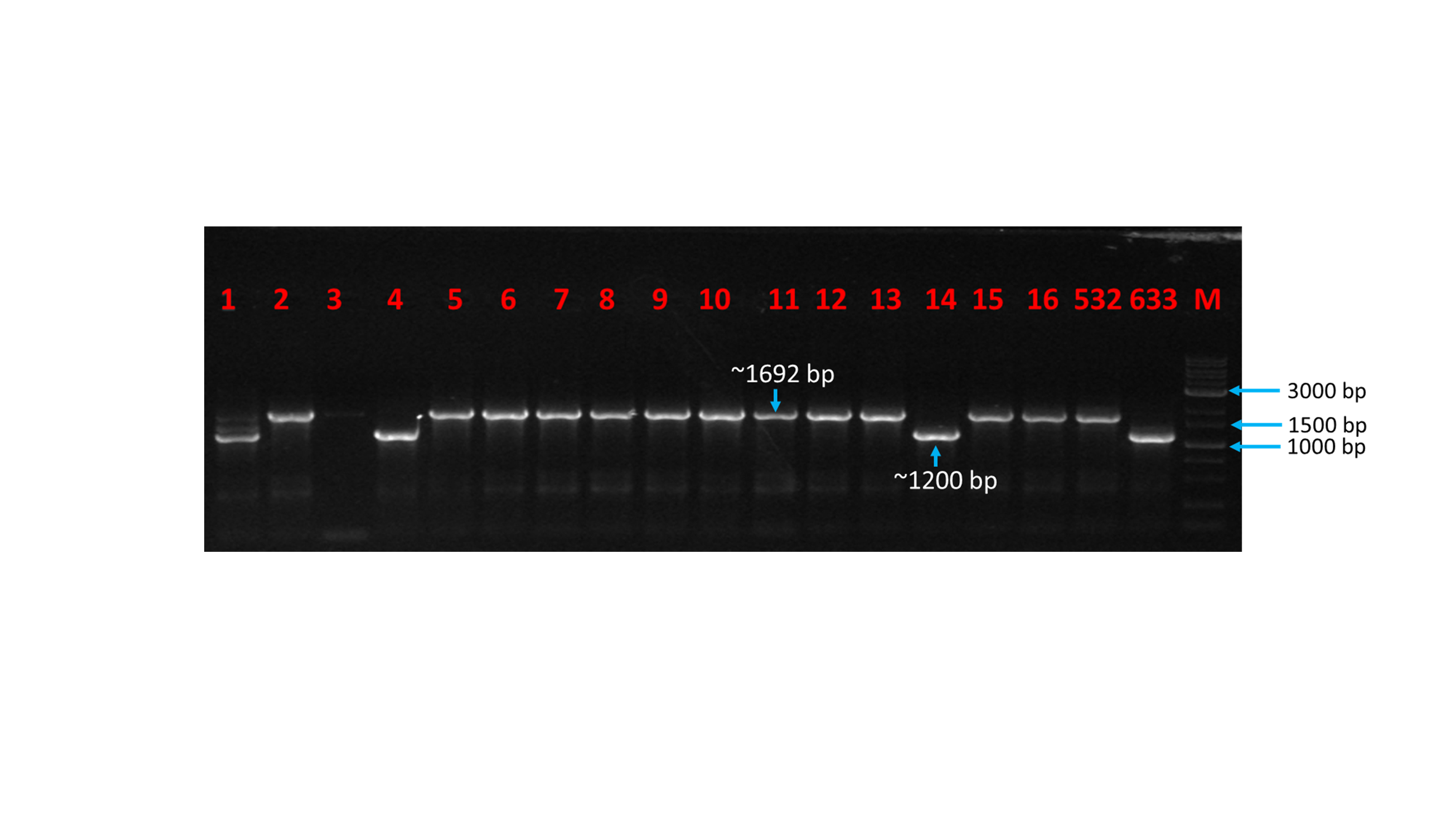

Supplement: uqaf047_Supplemental_Files [file uqaf047_supplemental_files.zip › Supplementary Fig. 3.tif]

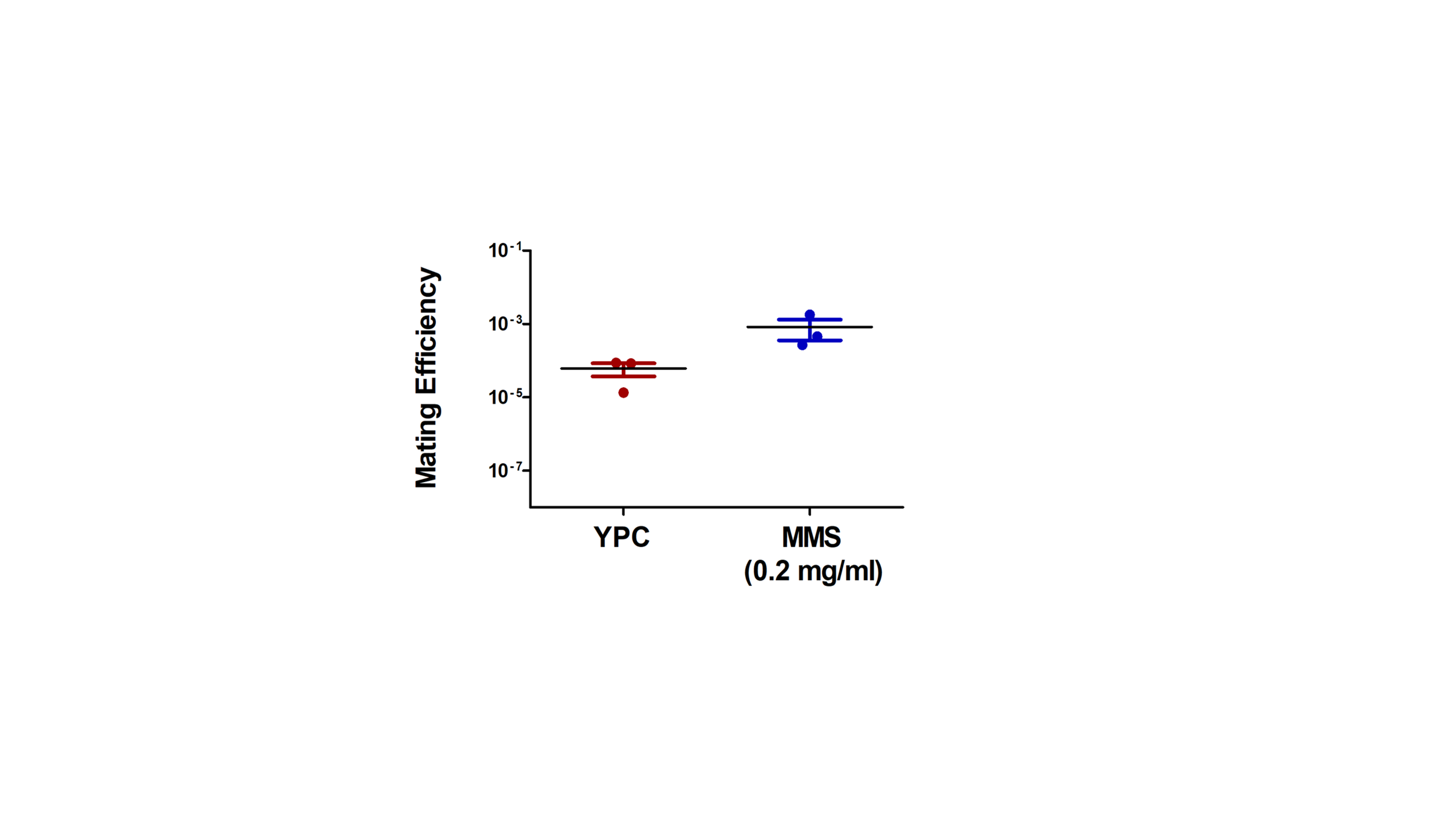

Supplement: uqaf047_Supplemental_Files [file uqaf047_supplemental_files.zip › Supplementary Fig. 4.tif]
